# Inhibitory Effects of Megakaryocytic Cells in Prostate Cancer Skeletal Metastasis

**DOI:** 10.1002/jbmr.204

**Published:** 2010-08-03

**Authors:** Xin Li, Amy J Koh, Zhengyan Wang, Fabiana N Soki, Serk In Park, Kenneth J Pienta, Laurie K McCauley

**Affiliations:** 1 Periodontics and Oral Medicine, University of Michigan School of DentistryAnn Arbor, MI, USA; 2 Department of Orthodontics and Pediatric Dentistry, University of Michigan School of DentistryAnn Arbor, MI, USA; 3 Department of Urology, University of Michigan Medical SchoolAnn Arbor, MI, USA; 4 Department of Pathology, University of Michigan Medical SchoolAnn Arbor, MI, USA

**Keywords:** MEGAKARYOCYTES, PROSTATE CARCINOMA, SKELETAL METASTASIS, APOPTOSIS

## Abstract

Prostate cancer cells commonly spread through the circulation, but few successfully generate metastatic foci in bone. Osteoclastic cellular activity has been proposed as an initiating event for skeletal metastasis. Megakaryocytes (MKs) inhibit osteoclastogenesis, which could have an impact on tumor establishment in bone. Given the location of mature MKs at vascular sinusoids, they may be the first cells to physically encounter cancer cells as they enter the bone marrow. Identification of the interaction between MKs and prostate cancer cells was the focus of this study. K562 (human MK precursors) and primary MKs derived from mouse bone marrow hematopoietic precursor cells potently suppressed prostate carcinoma PC-3 cells in coculture. The inhibitory effects were specific to prostate carcinoma cells and were enhanced by direct cell-cell contact. Flow cytometry for propidium iodide (PI) and annexin V supported a proapoptotic role for K562 cells in limiting PC-3 cells. Gene expression analysis revealed reduced mRNA levels for cyclin D1, whereas mRNA levels of apoptosis-associated specklike protein containing a CARD (ASC) and death-associated protein kinase 1 (DAPK1) were increased in PC-3 cells after coculture with K562 cells. Recombinant thrombopoietin (TPO) was used to expand MKs in the marrow and resulted in decreased skeletal lesion development after intracardiac tumor inoculation. These novel findings suggest a potent inhibitory role of MKs in prostate carcinoma cell growth in vitro and in vivo. This new finding, of an interaction of metastatic tumors and hematopoietic cells during tumor colonization in bone, ultimately will lead to improved therapeutic interventions for prostate cancer patients. © 2011 American Society for Bone and Mineral Research.

## Introduction

Prostate cancer is the most common malignancy in American men. It alone accounts for about 25% of cancer cases in men.(1) Bone metastasis is the major cause of mortality associated with prostate cancer and affects up to 90% of patients dying with advanced disease.(2) The classic concept that circulating tumor cells need “congenial soil” to “seed” has stimulated attention focused on understanding the dynamic migratory abilities of tumor cells.(3) Meanwhile, understanding the characteristics and early changes occurring in the bone marrow microenvironment that welcome incoming cancer cells is still lacking. Within the skeleton, prostate cancer often spreads to the axial skeleton and long bone metaphyses, sites under active remodeling that contain robust marrow cellularity.(4) Bone, as a dynamic and complicated system, is filled with rich bone marrow and blood vessels. There are two kinds of stem cells that reside in the bone marrow: hematopoietic stem cells (HSCs) and mesenchymal stem cells (MSCs). HSCs and their progeny are surrounded by stromal cells in the marrow. MSCs reside in the bone and give rise to the majority of marrow stromal cell lineages, including chondrocytes, osteoblasts, fibroblasts, adipocytes, endothelial cells, and myocytes.(5,6) Developing hematopoietic cells in the bone marrow are retained until they mature and are released into the circulation. That hematopoietic cells have been observed in close juxtaposition to osteoblasts (OBs) and that cytokines known to be important for hematopoiesis are expressed on the cell surfaces or secreted by OBs have lead scientists to explore the interaction between hematopoietic cells and cells of the marrow stromal cell lineage. Increased observations show that the osteoblastic niche is important in supporting and maintaining HSCs.(7) Megakaryocytopoiesis occurs in the hematopoietic (extravascular) compartment of the marrow; megakaryocytes (MKs) then migrate and locate parasinusoidally, the cytoplasm invaginates, penetrates the endothelial cell lining, and platelets are released into the blood circulation.(8) This process is regulated by the action of numerous factors, including cytokines, growth factors, chemokines, and extracellular matrix molecules (ECMs), many of which are produced by stromal cells within the marrow microenvironment. The development and proliferation of osteoblasts directly influences hematopoiesis(7,9–11) and megakaryopoiesis,(12,13) further illustrating the significance of these interactions.

Recently, MKs have been demonstrated to have inhibitory effects on osteoclast (OC) formation and activity.(14–17) These studies demonstrated that a factor or factors secreted by MKs inhibit OC development. The emerging paradigm is that MKs play a dual role in regulating skeletal homeostasis by simultaneously stimulating OB proliferation and differentiation and inhibiting OC development.(14–17) As a result, alterations in MK proliferation or differentiation also could affect skeletal changes. Considering that bone resorption is favorable to tumor growth in bone(18–20) and there is a high tendency of prostate cancer to localize in bone, MKs may indirectly inhibit tumor growth via decreased OC resorption and reduced cytokine and growth factor release from the bone matrix.(15–17) Another possibility is that MKs directly affect prostate cancer cells in their trajectory through the bone marrow microenvironment. However, investigations on the interaction between MKs and metastatic tumor cells are minimal. This study is based on the hypothesis that MKs inhibit prostate cancer cell growth in bone through direct inhibition of cancer cells and indirectly by their influence in the bone microenvironment.

## Materials and Methods

### Cell culture

Human hematopoietic precursor cells K562 (a patient-derived leukemia cell line expressing both erythroid and MK markers that can be induced to differentiate along each of these pathways) and Meg01 (a patient-derived megakaryoblastic cell line) were used as precursors for MKs. HL60 cells (a patient-derived promyelocytic leukemia cell line that can be differentiated to neutrophilic promyelocytes) were cultured in suspension under appropriate growth conditions. K562 cells were maintained in Iscove's Modified Dulbecco's Media (IMDM) + 10% fetal bovine serum (FBS) (Invitrogen Corp., Carlsbad, CA, USA) and induced with 50 nM of phorbol myristate acetate (PMA) (Sigma Chemical Co., St. Louis, MO, USA) to differentiate into mature MKs. To differentiate K562 cells to erythroid cells, 40 nM of doxorubicin hydrochloride (DOX, Sigma) was added to the culture medium of K562 cells for 3 days. Meg01 cells and HL60 cells were maintained in RPMI (Roswell Park Memorial Institute) 1640 media + FBS. Both cells were passaged at a density of about 2 × 10^5^/mL when seeding.

Primary MKs were isolated and cultured from mouse bone marrow cells. Briefly, after flushing and centrifugation with Ficoll-Paque (GE Healthcare, Piscataway, NJ, USA), bone marrow cells were harvested, counted, and seeded at 10^6^/mL with addition of nucleotide mixtures (Invitrogen), thrombopoietin (TPO), interleukin 6 (IL-6), and IL-11 (PeproTech, Rocky Hill, NJ, USA). Five or six days later, MKs were enriched by gravity sedimentation twice before use for coculture assay. The upper-portion cells were collected as less mature MKs and the bottom-layer cells were collected as mature MKs. The primary MK culture method was the same as described previously, where it was demonstrated that after 6 days of culture, the purity of MKs (both in medium-sized and large populations) was 98%.(21)

PC-3 prostate carcinoma cells stably expressing luciferase (PC-3^*Luc+*^, referred to as PC-3)(22) and C4-2b cells were maintained in RPMI 1640 + 10% FBS. The VCaP prostate carcinoma cell line was maintained in DMEM + 10% FBS. Cells were passaged using trypsin with EDTA (Invitrogen) and resuspended in appropriate growth medium.

Human osteoblastic SaOS2 cells were maintained and passaged every 4 to 5 days in α-MEM (Invitrogen) containing 100 units/mL of penicillin and streptomycin and 10% FBS. SaOS2 cells were plated at 50,000 cells/cm^2^ and cultured to 80% to 90% confluence.

### Viable cell enumeration

Five thousand (5 × 10^3^) cells (adherent prostate carcinoma cells or osteoblastic cells) were plated per well in 24-well plates in quadruplicate in the presence of serum at the indicated concentrations. K562 cells, HL60 cells, or MKs induced from K562 cells were seeded in the suspension medium over the adherent cells. On the indicated days, suspension cells were removed before PC-3 cells were washed and trypsinized. The luciferase activity of cell lysates as a reflection of cell number was determined with the dual luciferase reporter assay system (Promega, Madison, WI, USA) and a Monolight 2010 Luminometer (BD-Pharmingen, San Diego, CA, USA). The non-luciferase-expressing adherent cells were washed with PBS and fixed with 40% methanol, followed by staining with crystal violet. DNA binding dye was solubilized with 10% acetic acid. Relative cell numbers were measured by optical density at 595 nm.

### Protein isolation and analysis

Protein levels of apoptosis-associated specklike protein containing a CARD (ASC) and death-associated protein kinase 1 (DAPK1) were determined by Western blot using total cell lysates. Briefly, cells were washed with cold PBS and collected with Invitrogen SDS buffer. Samples were stored at −20°C until assays were performed. Western blots were performed using actin as a loading control in each sample (all antibodies were from Cell Signaling Technology, Danvers, MA, USA). Western blot films were scanned, and relative protein expression was quantified using the ImageJ analysis program.

### In vivo prostate cancer models

Four-week-old male athymic mice were obtained from Harlan (Indianapolis, IN, USA). Mice (20/group) were pretreated with TPO or vehicle for 5 days before inoculation of tumor cells. Five mice from each group were euthanized for histology and flow cytometry analyses using fluorescein isothiocyanate (FITC)–labeled CD41 antibody (BD-Pharmingen) to determine CD41^+^ cell numbers in the bone marrow to confirm the effect of TPO. The remaining mice were sedated with 1.7% isoflurane mixed with air, and 100,000 PC-3 cells were inoculated via intracardiac injection into the left ventricle, as described previously.(23) Mice were sacrificed after 4 weeks, and the skeletons were exposed to X-ray film (Wolverine X-Ray, Dearborn, MI, USA) at 32 kV for 45 seconds in a microradiography X-ray machine (Faxitron, Madison, WI, USA) to identify gross skeletal changes. Tibias were fixed in 10% formalin, decalcified, and processed for histologic analysis. All animal studies were approved by the University of Michigan Committee on the Use and Care of Animals (UCUCA).

### In vivo localization and growth of PC-3 prostate cancer cells

In vivo bioluminescent imaging was carried out at the University of Michigan Small Animal Imaging Resource facility. Before imaging, mice were injected i.p. with 100 µL of 40 mg/mL luciferin dissolved in PBS. Imaging was performed under 1.75% isoflurane/air anesthesia on a cryogenically cooled in vivo imaging system (IVIS) equipped with a 50-mm lens and coupled with a data-acquisition PC running Living Image Software 2.6 (Xenogen Corp., Alameda, CA, USA). Ventral images were acquired 12 minutes after injection. Pseudocolor images of photon emissions were overlaid on grayscale images of mice to aid in determining signal spatial distribution. Photon quantifications were calculated within regions of interest (ROI).

### Histology and immunohistochemistry

Xenograft tumors were harvested and placed in fresh 10% formalin. Tibias were decalcified in 10% EDTA for 21 days prior to paraffin embedding. Paraffin-embedded specimens were sectioned (5 µm) and stained with either hematoxylin and eosin (H&E), trichrome (bone), or tartate resistant acid phosphatase (TRACP) (osteoclasts; Acid Phosphatase, Leukocyte Kit, Sigma), or immunohistochemistry was performed for von Willebrand factor (vWF). Standard indirect immunoperoxidase procedures were used for immunohistochemistry using the AEC Cell and Tissue Staining System (R&D Systems, Minneapolis, MN, USA). Mayer's hematoxylin (Sigma) was used for counterstaining.

### Flow cytometric analysis for cell cycle and apoptosis

Cells were washed twice with cold PBS and lifted with trypsin for 3 minutes at 37°C before being stained with propidium iodide (PI) or the Annexin V-FITC Apoptosis Detection System (BD-Pharmingen). Cells also were treated with ribonuclease A before staining with PI for cell cycle analysis. FITC-conjugated annexin V and PI staining were evaluated with a FACs Calibur (BD Bioscience, San Jose, CA, USA).

### RNA isolation and mRNA quantification

RNA was isolated using Tri reagent (Sigma) following the manufacturer's protocols. One microgram of total RNA was reverse transcribed in a 20-µL reaction volume containing random hexamers using a reverse-transcription assay system (Applied Biosystems, Foster City, CA, USA). Semiquantitative RT-PCR was performed on a GeneAmp 7700 thermocyler (Applied Biosystems), and fold changes were determined via the ΔΔ*C*_*T*_ method with normalization to *GAPDH* mRNA levels.

### Statistical analysis

One-way ANOVA or the Student's *t* test for independent analysis was applied to evaluate differences, and Fisher's exact test was applied to compare the incidence rate of lesion development using the GraphPad Instat Software Program (GraphPad Software, Inc., San Diego, CA, USA). A value of *p* < .05 was considered statistically significant. All assays were repeated a minimum of two times with similar results.

## Results

### K562/MK cells inhibited prostate carcinoma cell growth in vitro

**Fig. 1:**
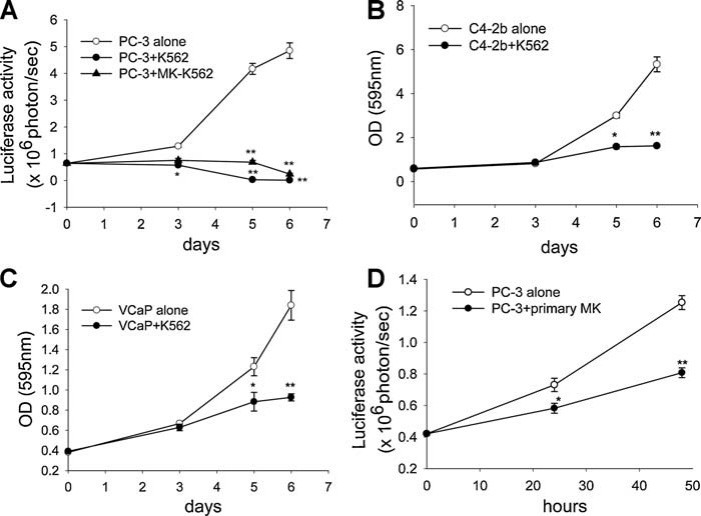
Prostate carcinoma cell growth is inhibited by K562/MK cells in vitro. Five thousand luciferase-tagged prostate carcinoma PC-3 cells (PC-3^*luc+*^, adherent, referred to as PC-3) were cocultured with 20,000 K562 cells (nonadherent) or mature MKs derived from K562 cells or mouse bone marrow cells (nonadherent, referred to as MK-K562 cells) for up to 6 days. MK cells were removed with the supernatant, and only adherent cells were analyzed. (*A*) PC-3 cell lysates were analyzed for luciferase activity using the luciferase assay reagent (Promega). VCaP (*B*) and C4-2B (*C*) cells were fixed and stained following a protocol using crystal violet. (*D*) Ten thousand PC-3 cells were cocultured with 10,000 mature primary MKs for up to 48 hours. PC-3 cell lysates were analyzed for luciferase activity using the luciferase assay reagent. All assays were performed in triplicate. *p < .05; **p < .005 versus PC-3 alone.

The K562 cell line is an established precursor cell line of MKs and erythroid cells on differential stimulation. When induced by PMA, K562 cells differentiate into MKs accompanied by a net increase in megakaryocytic markers and a reduction in erythroid markers.(24) K562 cells, with or without PMA pretreatment, were cocultured with the prostate carcinoma cell line PC-3 previously labeled with a luciferase tag. Strong inhibition of PC-3 growth was found in both conditions (Fig. 1*A*). This inhibitory effect of K562 on prostate carcinoma growth was further confirmed using two other prostate carcinoma cell lines, VCaP and C4-2B cells (Fig. 1*B, C*). In addition, primary MKs derived from mouse bone marrow also cells demonstrated significant inhibition of PC-3 cell growth in coculture (Fig. 1*D*). The primary megakaryocytes used in coculture were fairly pure (>90%) after gravity sedimentation, as measured by flow cytometry using CD41 as the marker for MK (Supplemental Fig. 1).

### K562/MK inhibition of cell growth is specific to prostate carcinoma and enhanced by close proximity

**Fig. 2:**
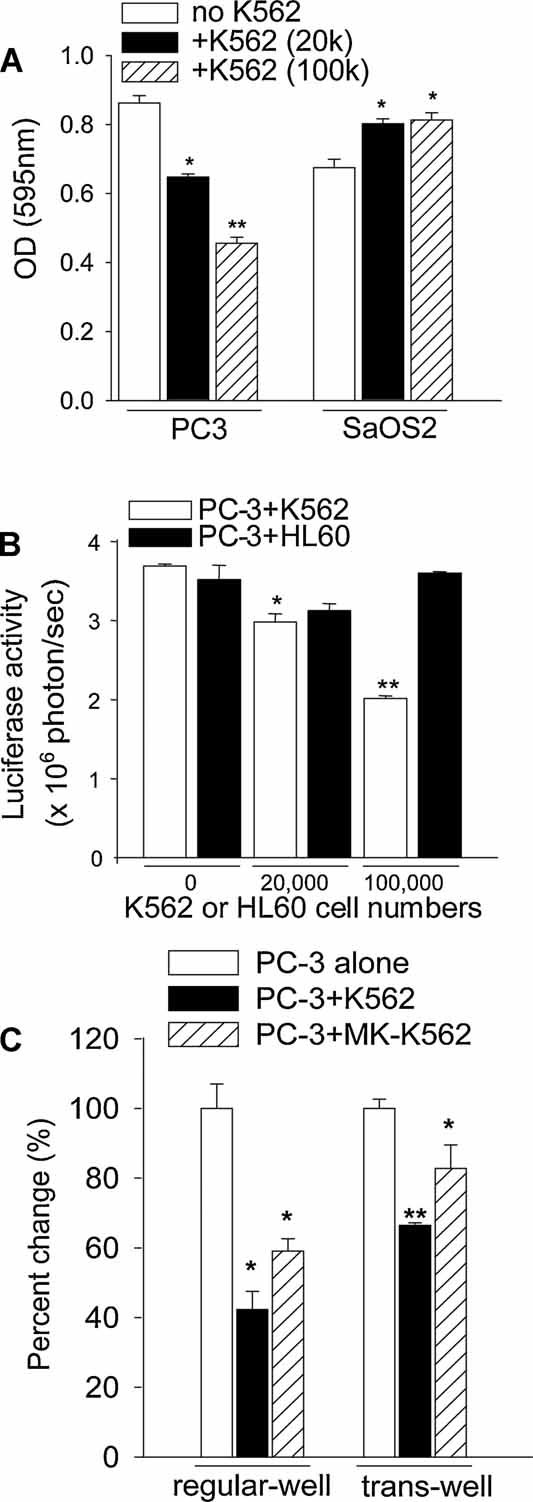
MK inhibition of cell growth is specific to prostate carcinoma and enhanced by close proximity. (*A*) Growth of PC-3 cells was inhibited, whereas human osteoblastic cell (SaOS2) growth was upregulated by K562 cells after 3 days of coculture. (*B*) K562 cells but not HL60 cells inhibited the growth of PC-3 cells after 3 days of coculture. K562 and HL60 cells were removed with supernatant, and only adherent cells were left for the analysis. (*C*) Five thousand PC-3 cells were cultured in the upper chambers with 20,000 K562 cells or MKs differentiated from K562 cells in the bottom wells for 3 days. After three washes with PBS, PC-3 cell lysates were analyzed immediately for luciferase activity (data shown as percent change versus luciferase activity of PC-3 cells alone). All assays were performed in triplicate. *p < .05; **p < .005 versus PC-3 or SaOS2 cells alone.

To examine the specificity of MK inhibition of prostate carcinoma cell growth, cocultures with nonepithelial SaOS2 cells, a human osteosarcoma cell line, and HL60, a human promyelocytic leukemia cell line, were performed. Unlike the inhibitory effects seen with prostate carcinoma cells, a significant stimulation of SaOS2 cell growth was observed (Fig. 2*A*). Furthermore, HL60 failed to regulate the growth of prostate carcinoma (Fig. 2*B*). To evaluate the dependence of direct cell-cell contact on the inhibitory effects of prekaryocytes and MKs, a transwell culture system was used, in which PC-3 cells and K562 cells or MKs were cultured separately in the upper and lower wells, respectively. The inhibitory effect of MKs on PC-3 cells was still present but was significantly blunted when K562 cells or MKs differentiated from K562 cells (MK-K562 cells) were cultured with PC-3 cells in the transwell system without direct contact (Fig. 2*C*). Without very close proximity or cell-cell contact, K562 cells still blocked the growth of PC-3 cells by 35% (transwell plate), whereas mature MKs only inhibited PC-3 cell growth by 15% in transwell plates versus 55% in regular-well coculture. This suggests that a very close proximity or direct cell-cell contact is important for the maximal inhibitory effects of mature MKs. Thus either a very steep inhibitory cytokine/growth factor gradient that requires very close proximity or a primarily membrane-bound factor that requires direct cell-cell contact to exert the inhibitory effects exists.

### Primary megakaryocytes inhibited PC-3 cell growth regardless of maturation phase

**Fig. 3:**
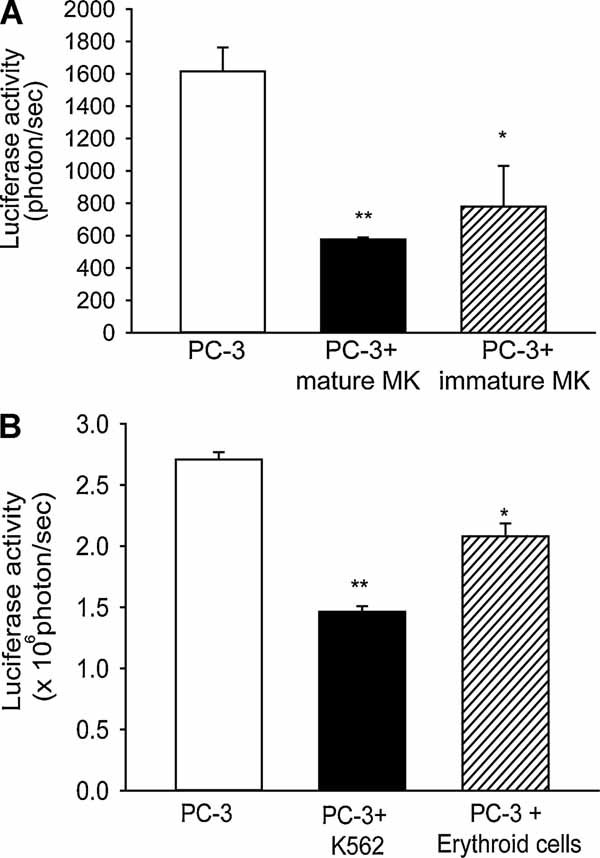
MKs but not erythroid cells inhibited PC-3 cell growth. (*A*) Ten thousand PC-3 cells were cocultured with 10,000 primary mature (larger size CD41^+^) or less mature (immature, smaller size CD41^+^) MKs for 3 days. (*B*) Ten thousand PC-3 cells were cocultured with 10,000 erythroid cells derived from K562 cells after doxorubicin hydrochloride (DOX) treatment (40 nM). In both experiments, PC-3 cells were washed twice with PBS, and the cell lysates were collected with passive lysis buffer and analyzed for luciferase activity and expressed as photons per second. Assays were performed in triplicate. *p < .05; **p < .001 versus PC3 cells alone.

Since the inhibitory effect of K562 cells was more potent than that of mature MKs derived from K562 cells, primary MKs cultured from mouse bone marrow were used to determine whether maturation of MKs affects their inhibitory ability. Primary MKs at different mature phases were cultured with PC-3 cells for 3 days. Both populations suppressed PC-3 cell growth without significant differences (Fig. 3*A*). Erythroid cells derived from K562 cells also were tested for the effects on prostate carcinoma cell growth in a coculture system (Fig. 3*B*). Unlike MKs derived from K562 cells, erythroid cells differentiated from K562 cells inhibited PC-3 cell growth by only 20%. Considering that the 3-day 40 nM of DOX treatment can only partially differentiate K562 cells to erythroid cells (Supplemental Fig. 2,^25^) it is likely that the 20% inhibitory effects actually were due to the remaining K562 cells in the culture, and the influence of erythroid cells was minimal.

### K562/MKs induced apoptosis in PC-3 cells

**Fig. 4:**
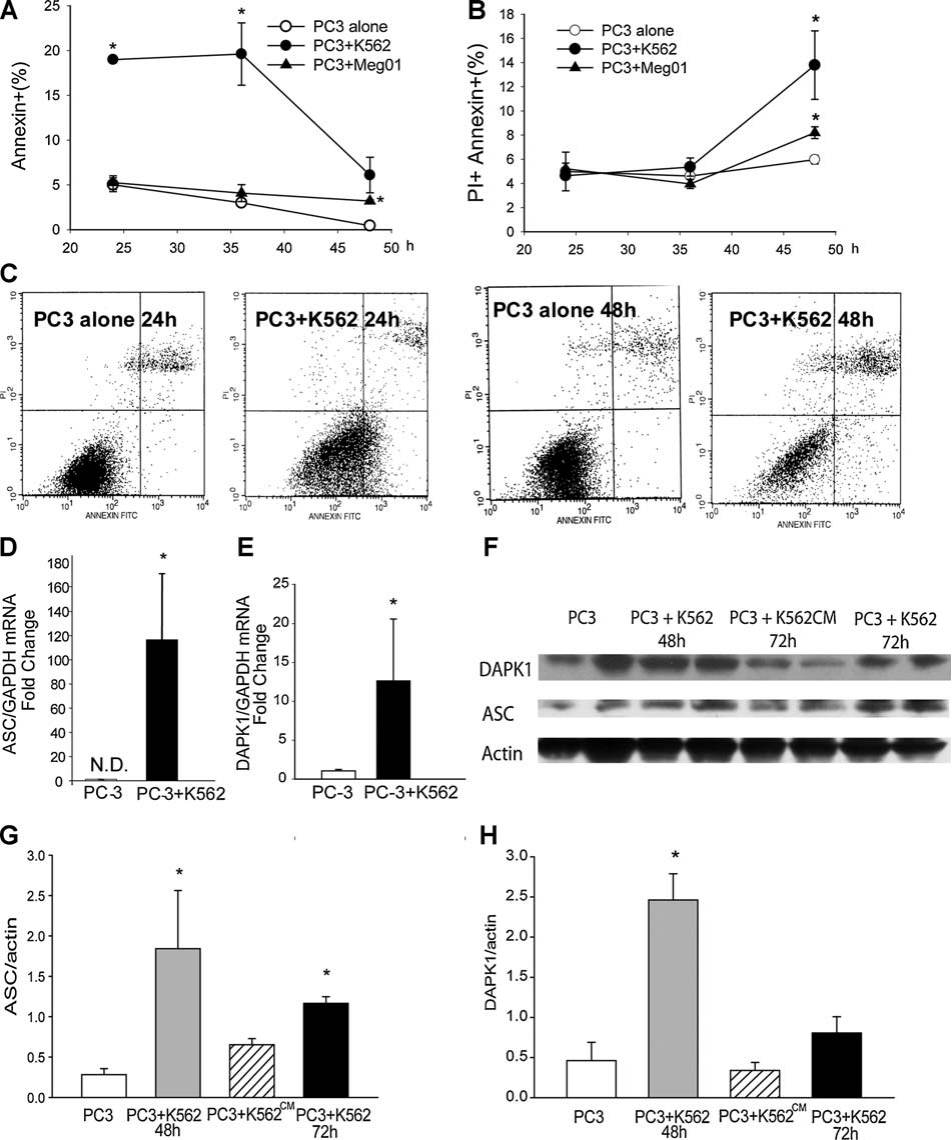
MK cells increased apoptosis and ASC and DAPK1 expression in PC-3 cells. PC-3 cells were cocultured with the MK progenitor cell line K562 or Meg01 cells for 24, 36, and 48 hours before annexin V and propidium iodide staining. (*A*) Annexin V^+^ and (*B*) both annexin V^+^ and PI^+^ cell populations in PC-3 cells after coculture with K562 cells or Meg01 cells. (*C*) Representative fluorescence-activated cell-sorting (FACS) images of PC-3 cells cultured with or without K562 cells at 24 and 48 hour. qPCR using Carboxyfluorescein (FAM)-labeled primers for *ASC* (*D*) and *DAPK1* (*E*) mRNA levels. (*F*) Representative Western blot of ASC and DAPK1 protein detection in PC-3 cells cultured with K562 cells for 48 and 72 hours. Quantitative protein levels of ASC (*G*) and DAPK1 (*H*). All assays were performed in triplicate. N.D.= nondetectable. *p < .05; **p < .001 versus PC-3 alone.

To understand the mechanism of the inhibitory effects of MKs on prostate carcinoma cell growth, the MK and PC-3 coculture system was evaluated with cell-cycle and apoptosis assays. Flow cytometric analysis demonstrated that K562 and MK-K562 cells altered the ratios of PC-3 cells in cell-cycle phases (Supplemental Fig. 3*A*). The percentage of cells in G_0_ /G_1_ phase increased, whereas cells in S and G_2_ /M phases decreased by coculture with K562 and MK-K562 cells. It was further demonstrated by quantitative polymerase chain reaction (qPCR) that there was a 50% decrease in PC-3 *cyclin D1* mRNA levels after 48 hours of coculture with K562 cells (Supplemental Fig. 3*B*); however, these changes were not enough to decrease PC-3 cell growth to the extent observed. K562 cells and another MK precursor cell, Meg01 cells, also significantly induced apoptosis of PC-3 cells. A dramatic increase in annexin V^+^ cell (early apoptotic cells) number was observed in PC-3 cells after 24 and 36 hours of coculture with K562 cells. A significant increase in early apoptotic cells was observed in PC-3 cells cultured with Meg01 cells at 48 hours (Fig. 4*A*). This was followed by a significant increase in both annexin V^+^ and PI^+^ cell populations (late apoptotic cells) in PC-3 cells after 48 hours of coculture with K562 cells and Meg01 cells (Fig. 4*B*). An apoptosis pathway analysis (Supplemental Table 1) revealed *ASC* and *DAPK1* as two highly stimulated genes in PC-3 cells after coculture with K562 cells. The significant induction of both *ASC* and *DAPK1* mRNA levels was confirmed with qPCR (Fig. 4*D, E*). The protein levels of both ASC and DAPK1 were increased in PC-3 cells after 48 hours of incubation with K562 cells (Fig. 4*F–H*). Until 72 hours, ASC protein levels still were significantly higher than control.

### Increased MKs in vivo were associated with reduced prostate skeletal metastatic lesions

**Fig. 5:**
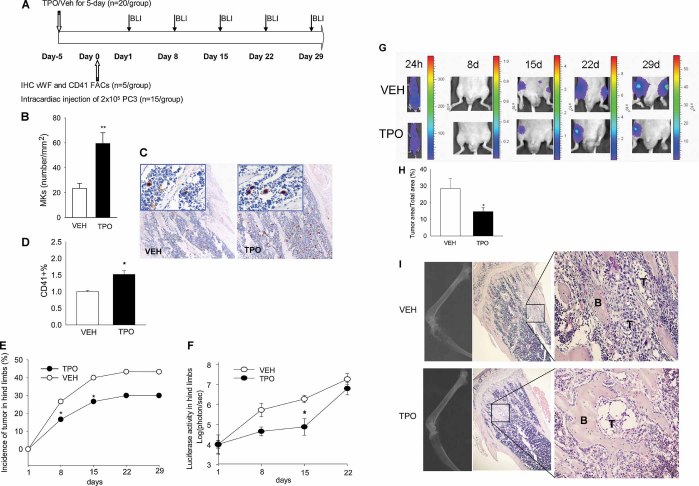
Expansion of MKs in vivo decreased prostate tumor metastatic lesion development. (*A*) Schematic of experimental design: Mice (20/group) were treated with recombinant TPO intraperitoneally to increase MKs. One day after the last injection, 5 mice from each group were euthanized, and one tibia was collected for (*B*) vWF immunohistochemistry and (*C*) representative images of vWF staining of the tibia sections from vehicle- and TPO-treated groups before tumor injection. (*C*) The other hind limb was flushed with PBS for flow cytometric staining of CD41^+^ MK cells. (*E*, *F*) The remaining mice were injected via the intracardiac route with 200,000 PC-3 cells, and tumor cell location and activity were recorded with weekly bioluminescence (BLI) imaging. The location and progress (*E*) of inoculated prostate carcinoma cells in mice were recorded with BLI imaging weekly, and the incidence rate of tumor in the legs (*F*) was enumerated weekly and analyzed by Fisher's exact test (*p < .05 versus vehicle). (*G*) Representative images of BLI of tumor development in hind limbs of vehicle- (VEH) and TPO-treated groups. (*H*) Histomorphometric analysis of tumor area in the tibias was decreased in mice pretreated with TPO. (*I*) Representative radiographs of hind limb and H&E staining of the tibial sections from vehicle- and TPO-treated groups 4 weeks after tumor injection. T = tumor; B = bone. *p < .05; **p < .01 versus VEH.

Thrombopoietin (TPO), the major MK growth factor is required for MK proliferation and maturation and was used to expand MKs to determine their impact on tumor metastasis in vivo (Fig. 5*A*). An increase in MKs after TPO priming was confirmed by histologic staining for the MK marker von Willebrand factor (vWF) and flow cytometric analysis of CD41^+^ cells in the bone marrow of a subset of athymic mice from each group 1 day after the last injection of TPO or vehicle (veh) and before cardiac inoculations (Fig. 5*B–D*). The incidence of lesion development in the legs after intracardiac inoculation of PC-3 cells was significantly reduced in mice pretreated with TPO (Fig. 5*E*). Meanwhile, the bioluminescence (BLI) activity of bone lesions in the legs of TPO-treated mice was less than in the vehicle-treated group at 2 weeks after tumor cell inoculation (Fig. 5*F*). Consistent with these live-image results, histomorphometric analysis indicated that the tumor area in the tibias of mice pretreated with TPO at euthanization was significantly less than in the controls (Fig. 5*G–I*).

**Fig. 6:**
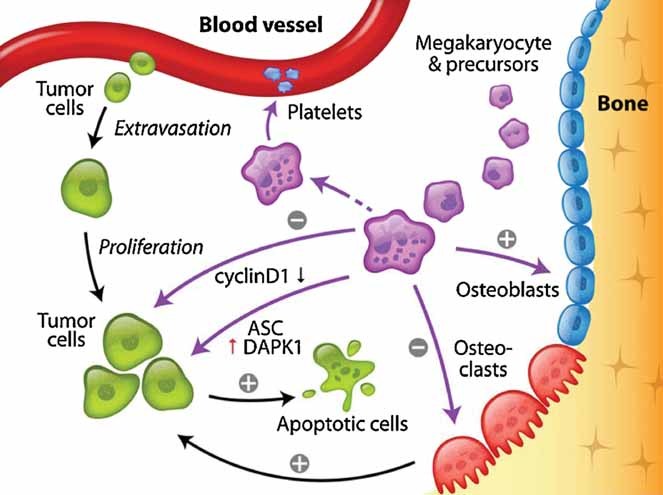
A schematic model to summarize the findings. MKs inhibit prostate cancer cell growth in bone through direct inhibition of cancer cells and indirectly by decreasing bone remodeling. The direct inhibition involves a marginal cell-cycle arrest via reduction in cyclin D1 and significant stimulation of apoptosis via induction of ASC and DAPK1. Meanwhile, MKs exert an anabolic effect in bone by stimulating osteoblasts and inhibiting osteoclasts.(16,17)

Based on these results, a schematic model was proposed to summarize the findings (Fig. 6). MKs inhibit prostate cancer cell growth in bone through direct induction of cancer cell apoptosis and indirectly, as described by others, by decreasing bone remodeling.(16,17)

## Discussion

In this study, K562 cells and MKs derived either from K562 cells or mouse bone marrow cells suppressed prostate carcinoma cell growth, whereas erythroid cells derived from K562 cells and another leukemia cell line, HL60, which differentiates along the macrophage or neutrophil lineage, had negligible effects. These results revealed a novel and specific inhibitory effect of MKs originated from bone marrow hematopoietic progenitor cells on prostate carcinoma cell growth in vitro and in vivo. To our knowledge, this is the first report of a specific MK-mediated inhibition of prostate cancer cell growth and contributes to the understanding of the interaction among cancer cells and native cells in the bone microenvironment. In addition, the location of mature MKs at the parasinusoids may make them the first defensive cells in the bone marrow facing tumor cells in their extravascular process.

Bone turnover contributes to tumor localization, and bisphosphonates, which suppress bone turnover, have been used effectively for the management of cancer-induced skeletal metastasis.(4,26–28) Since MKs potently inhibit osteoclastogenesis in vitro,(16) expansion of MKs with TPO may result in reduced osteoclast activity in vivo, which also inhibits prostate carcinoma cell growth in the bone marrow. Hence the decrease in prostate tumor bone lesion development in mice pretreated with TPO could be the combined effect of altered OCs (indirect) and MK cells (direct). However, in this study, the direct effects may be the major ones because the reduction in OC activity was mild and not significant after 5 days of TPO treatment (Supplemental Fig. 4).

The inhibitory effects of MKs on prostate carcinoma were not sustained over time in vivo because the luciferase activity of the tumor cells in the hind limbs of the TPO-treated mice caught up with that of the vehicle-treated mice at 3 weeks after tumor inoculation. These data may imply that the inhibitory effects from MKs and their precursors are early events taking place during tumor cell seeding in the marrow. During this period, MKs have advantages over the tumor cells in regard to cell numbers and/or density. Once the tumor cells exit the blood vessels and enter the marrow, they may be under limited influence of MKs because there may be less opportunity for their encounter.

It has been reported that granulocyte colony-stimulating factor (G-CSF) treatment enhanced bone tumor growth in mice.(29) Both G-CSF and TPO may increase bone marrow cellularity, but since TPO treatment reduced tumor growth and G-CSF increased tumor growth in bone, this suggests that the decrease in skeletal tumor burden in TPO-treated mice was less likely due to the general changes in bone marrow cellularity.

MKs produce platelets, which have been shown to support the metastatic potential of tumor cells, and inhibiting platelet aggregation decreases tumor metastasis.(30,31) It is possible that with the expansion of MKs in the bone marrow, the number of platelets increased, which favored tumor growth later and hence a reduction in tumor protection with time after TPO treatment. Interestingly, the size of platelets is reduced in patients with solid tumors and skeletal metastasis.(32) Although the mechanism underlying this observation is obscure, it is known that platelet size is determined at the MK stage and is inversely proportional to MK ploidy.(33) MK ploidy is regulated by cytokines such as IL-3 and IL-6, which contribute to more reactive and larger platelets.(33,34) Identifying the interaction between tumor cells and MKs could result in a better understanding of the decrease in platelet size in patients with skeletal metastasis.

In mice, PC-3 cells have a tendency to metastasize to the jaw when they are injected into the circulation. Interestingly, in contrast to the long bones, tumor development in the jaws was similar between TPO- and vehicle-treated groups with intracardiac inoculation of PC-3 cells (Supplemental Fig. 5). Comparing the jaw with the long bones, there is a smaller marrow cavity; thus the structure of the jaw sets a limitation on the marrow cavity, as well as reduced numbers of MKs. The continuous tooth eruption of rodents and high bone turnover rates also may contribute to tumor development in the jaw. Hence the inhibitory effects of MKs in this location may be overruled by other factors in this model system.

Our data indicated that K562 cells suppressed the growth of prostate carcinoma cells mainly through enhancing apoptosis. Further experiments revealed that two apoptotic genes, *ASC* and *DAPK1*, were highly regulated genes associated with the inhibitory effects during coculture. *ASC*, as indicated by its name, apoptosis-associated specklike protein containing a CARD, contains a caspase recruitment domain (CARD), suggesting a role in caspase-mediated cell death. It has been shown that in breast cancers, ASC-induced apoptosis proceeds through a CARD-dependent aggregation step followed by activation of a caspase-9-mediated pathway.(35) Interestingly, both *ASC* and *DAPK1* gene promoters are reported to be hypermethylated in prostate carcinoma, including the PC-3 cell line.(36–39) Methylation in the promoter often involves loss of function of a gene and thus plays an essential role in maintaining normal cell function. Aberrant DNA methylation patterns may be the earliest somatic genome changes in prostate cancer. Changes in methylation contribute to multiple cancer development.(40,41) This study revealed a significant increase in ASC and DAPK1 expression in PC-3 cells after coculture with K562 cells, suggesting that K562 may induce PC-3 cell apoptosis through increasing the abundance of these genes normally deregulated in the process of carcinogenesis.

In summary, the original finding of megakaryocytic cell inhibitory effects on prostate carcinoma cells unmasked a novel osteoimmunologic response to the invasion of prostate carcinoma cells during skeletal metastasis. Moreover, discovery of the downstream mediators, ASC and DAPK1, provided new potential therapeutic targets for prostate cancer.

## References

[1] Jemal A, Murray T, Ward E (2005). Cancer statistics, 2005. CA Cancer J Clin..

[2] Rubin MA, Putzi M, Mucci N (2000). Rapid (“warm”) autopsy study for procurement of metastatic prostate cancer. Clin Cancer Res..

[3] Paget S (1989). The distribution of secondary growths in cancer of the breast. Cancer Metastasis Rev..

[4] Schneider A, Kalikin LM, Mattos AC (2005). Bone turnover mediates preferential localization of prostate cancer in the skeleton. Endocrinology..

[5] Wilson A, Trumpp A (2006). Bone-marrow haematopoietic stem-cell niches. Nat Rev Immunol..

[6] Yin T, Li L (2006). The stem cell niches in bone. J Clin Invest..

[7] Calvi LM, Adams GB, Weibrecht KW (2003). Osteoblastic cells regulate the haematopoietic stem cell niche. Nature..

[8] Lichtman MA, Chamberlain JK, Simon W, Santillo PA (1978). Parasinusoidal location of megakaryocytes in marrow: a determinant of platelet release. Am J Hematol..

[9] Kaplan RN, Riba RD, Zacharoulis S (2005). VEGFR1-positive haematopoietic bone marrow progenitors initiate the pre-metastatic niche. Nature..

[10] Liao J, McCauley LK (2006). Skeletal metastasis: Established and emerging roles of parathyroid hormone related protein (PTHrP). Cancer Metastasis Rev..

[11] Udagawa N, Takahashi N, Jimi E (1999). Osteoblasts/stromal cells stimulate osteoclast activation through expression of osteoclast differentiation factor/RANKL but not macrophage colony-stimulating factor: receptor activator of NF-kappa B ligand. Bone..

[12] Ahmed N, Khokher MA, Hassan HT (1999). Cytokine-induced expansion of human CD34+ stem/progenitor and CD34+CD41+ early megakaryocytic marrow cells cultured on normal osteoblasts. Stem Cells..

[13] Cheng L, Qasba P, Vanguri P, Thiede MA (2000). Human mesenchymal stem cells support megakaryocyte and pro-platelet formation from CD34(+) hematopoietic progenitor cells. J Cell Physiol..

[14] Bord S, Frith E, Ireland DC, Scott MA, Craig JI, Compston JE (2005). Megakaryocytes modulate osteoblast synthesis of type-l collagen, osteoprotegerin, and RANKL. Bone..

[15] Kacena MA, Gundberg CM, Horowitz MC (2006). A reciprocal regulatory interaction between megakaryocytes, bone cells, and hematopoietic stem cells. Bone..

[16] Kacena MA, Nelson T, Clough ME (2006). Megakaryocyte-mediated inhibition of osteoclast development. Bone..

[17] Kacena MA, Shivdasani RA, Wilson K (2004). Megakaryocyte-osteoblast interaction revealed in mice deficient in transcription factors GATA-1 and NF-E2. J Bone Miner Res..

[18] Li X, Loberg R, Liao J (2009). A destructive cascade mediated by CCL2 facilitates prostate cancer growth in bone. Cancer Res..

[19] Mundy GR (1997). Mechanisms of bone metastasis. Cancer..

[20] Roato I, D'Amelio P, Gorassini E (2008). Osteoclasts are active in bone forming metastases of prostate cancer patients. PLoS ONE..

[21] Shiraga M, Ritchie A, Aidoudi S (1999). Primary megakaryocytes reveal a role for transcription factor NF-E2 in integrin alpha IIb beta 3 signaling. J Cell Biol..

[22] Kalikin LM, Schneider A, Thakur MA (2003). In vivo visualization of metastatic prostate cancer and quantitation of disease progression in immunocompromised mice. Cancer Biol Ther..

[23] Liao J, Schneider A, Datta NS, McCauley LK (2006). Extracellular calcium as a candidate mediator of prostate cancer skeletal metastasis. Cancer Res..

[24] Jacquel A, Herrant M, Defamie V (2006). A survey of the signaling pathways involved in megakaryocytic differentiation of the human K562 leukemia cell line by molecular and c-DNA array analysis. Oncogene..

[25] Czyz M, Szulawska A, Bednarek AK, Duchler M (2005). Effects of anthracycline derivatives on human leukemia K562 cell growth and differentiation. Biochem Pharmacol..

[26] Guise TA, Kozlow WM, Heras-Herzig A, Padalecki SS, Yin JJ, Chirgwin JM (2005). Molecular mechanisms of breast cancer metastases to bone. Clin Breast Cancer..

[27] Michigami T, Hiraga T, Williams PJ (2002). The effect of the bisphosphonate ibandronate on breast cancer metastasis to visceral organs. Breast Cancer Res Treat..

[28] Saad F, Gleason DM, Murray R (2004). Long-term efficacy of zoledronic acid for the prevention of skeletal complications in patients with metastatic hormone-refractory prostate cancer. J Natl Cancer Inst..

[29] Hirbe AC, Uluckan O, Morgan EA (2007). Granulocyte colony-stimulating factor enhances bone tumor growth in mice in an osteoclast-dependent manner. Blood..

[30] Palumbo JS, Barney KA, Blevins EA (2008). Factor XIII transglutaminase supports hematogenous tumor cell metastasis through a mechanism dependent on natural killer cell function. J Thromb Haemost..

[31] Uluckan O, Eagleton MC, Floyd DH (2008). APT102, a novel adpase, cooperates with aspirin to disrupt bone metastasis in mice. J Cell Biochem..

[32] Aksoy S, Kilickap S, Hayran M (2008). Platelet size has diagnostic predictive value for bone marrow metastasis in patients with solid tumors. Int J Lab Hematol..

[33] Burstein SA (1994). Platelets and cytokines. Curr Opin Hematol..

[34] Peng J, Friese P, George JN, Dale GL, Burstein SA (1994). Alteration of platelet function in dogs mediated by interleukin-6. Blood..

[35] McConnell BB, Vertino PM (2000). Activation of a caspase-9-mediated apoptotic pathway by subcellular redistribution of the novel caspase recruitment domain protein TMS1. Cancer Res..

[36] Collard RL, Harya NS, Monzon FA, Maier CE, O'Keefe DS (2006). Methylation of the ASC gene promoter is associated with aggressive prostate cancer. Prostate..

[37] Das PM, Ramachandran K, Vanwert J (2006). Methylation mediated silencing of TMS1/ASC gene in prostate cancer. Mol Cancer..

[38] Neuhausen A, Florl AR, Grimm MO, Schulz WA (2006). DNA methylation alterations in urothelial carcinoma. Cancer Biol Ther..

[39] Yegnasubramanian S, Kowalski J, Gonzalgo ML (2004). Hypermethylation of CpG islands in primary and metastatic human prostate cancer. Cancer Res..

[40] Gray SG, Eriksson T, Ekstrom TJ (1999). Methylation, gene expression and the chromatin connection in cancer. Int J Mol Med..

[41] Vaid M, Floros J (2009). Surfactant protein DNA methylation: a new entrant in the field of lung cancer diagnostics?. Oncol Rep..

